# Flax rust infection transcriptomics reveals a transcriptional profile that may be indicative for rust *Avr* genes

**DOI:** 10.1371/journal.pone.0226106

**Published:** 2019-12-12

**Authors:** Wenjie Wu, Adnane Nemri, Leila M. Blackman, Ann-Maree Catanzariti, Jana Sperschneider, Gregory J. Lawrence, Peter N. Dodds, David A. Jones, Adrienne R. Hardham

**Affiliations:** 1 Division of Plant Sciences, Research School of Biology, The Australian National University, Canberra, Australia; 2 CSIRO Agriculture and Food, Canberra, Australia; 3 Biological Data Science Institute, the Australian National University, Canberra, Australia; Leibniz-Institut fur Naturstoff-Forschung und Infektionsbiologie eV Hans-Knoll-Institut, GERMANY

## Abstract

Secreted effectors of fungal pathogens are essential elements for disease development. However, lack of sequence conservation among identified effectors has long been a problem for predicting effector complements in fungi. Here we have explored the expression characteristics of avirulence (*Avr*) genes and candidate effectors of the flax rust fungus, *Melampsora lini*. We performed transcriptome sequencing and real-time quantitative PCR (qPCR) on RNA extracted from ungerminated spores, germinated spores, isolated haustoria and flax seedlings inoculated with *M*. *lini* isolate CH5 during plant infection. Genes encoding two categories of *M*. *lini* proteins, namely Avr proteins and plant cell wall degrading enzymes (CWDEs), were investigated in detail. Analysis of the expression profiles of 623 genes encoding predicted secreted proteins in the *M*. *lini* transcriptome shows that the six known *Avr* genes (*i*.*e*. *AvrM* (*avrM*), *AvrM14*, *AvrL2*, *AvrL567*, *AvrP123* (*AvrP*) and *AvrP4*) fall within a group of 64 similarly expressed genes that are induced *in planta* and show a peak of expression early in infection with a subsequent decline towards sporulation. Other genes within this group include two paralogues of *AvrL2*, an *AvrL567* virulence allele, and a number of genes encoding putative effector proteins. By contrast, *M*. *lini* genes encoding CWDEs fall into different expression clusters with their distribution often unrelated to their catalytic activity or substrate targets. These results suggest that synthesis of *M*. *lini* Avr proteins may be regulated in a coordinated fashion and that the expression profiling-based analysis has significant predictive power for the identification of candidate *Avr* genes.

## Introduction

Rust fungi, in the Basidiomycete order Pucciniales (formerly Uredinales), constitute the largest subgroup within the fungal kingdom [1]. There are now over 8,000 known species, the vast majority of which are plant pathogens [2, 3]. Rust fungi occur in a wide diversity of habitats and infect angiosperms, gymnosperms and ferns, including many plants that are important in agriculture, horticulture and forestry. As a group, their host range is extensive, but individual rust species are highly specific for infecting particular host plants [4]. Rust fungi cause severe diseases in cereals (including wheat, barley, oats and corn), sugar cane, forage and range grasses, beans, soybeans, peanuts, coffee, poplar and pine. Of major concern is the Ug99 strain of wheat stem rust currently found in Africa and the Middle East [5]. This isolate is virulent on 90% of the wheat varieties under cultivation and thus its potential spread seriously threatens food production worldwide.

Rust fungi are obligate, biotrophic pathogens that require living host plants for their growth, development and reproduction [1]. They have complex life cycles that can involve multiple host species and the production of multiple spore types. Repeated infection of the same host species by asexual urediniospores gives rise to huge numbers of spores which are passively dispersed in the wind and initiate disease, often on an epidemic scale [1, 3].

The infection cycle initiated by urediniospores on the leaf surface involves spore attachment and germination, and formation of an appressorium, an infection structure that enables leaf penetration via stomatal openings [6]. Intercellular growth of infection hyphae leads to contact with mesophyll cells and development of specialized infection structures called haustoria. Haustoria form after penetration of the mesophyll cell wall, invaginating the plant plasma membrane as they expand. The plant plasma membrane is not ruptured and the haustorial cytoplasm remains separated from the cytoplasm of the living plant cell by the haustorial plasma membrane, haustorial cell wall, an extrahaustorial matrix and the plant plasma membrane, termed the extrahaustorial membrane [1]. Haustoria play key roles in nutrient uptake from the host plant and in secretion of effector proteins critical for successful infection [1, 7, 8].

Fungal and oomycete haustoria and infection hyphae secrete hundreds, if not thousands, of distinct proteins [9–13], some of which are effectors. Some effectors have been shown to play roles in suppressing host defense responses or in orchestrating changes in host metabolism that favor pathogen development [14, 15], but the function of most candidate effectors is still not clear at the moment. Fungal effectors usually have little or no sequence homologies to other proteins and no shared sequence motifs have been characterized that might enable their identification *in silico* [16, 17]. Some effectors, such as CWDEs, function in the plant apoplast while others cross the plant plasma membrane and function in the plant cytoplasm [18, 19].

In the past, the molecular details of the infection process have been difficult to study for rust fungi due to their biotrophic life-style and the inability to obtain pure fungal samples in the absence of contaminating plant material. However, analysis of an infection transcriptome can yield information on patterns of pathogen gene expression during disease development which can, in turn, provide valuable clues for the identification of pathogen proteins that play important roles in pathogenicity [20–24]. Within the range of next-generation techniques, the RNA-Seq approach is especially powerful and has revolutionized transcriptome studies [25, 26].

One factor that is crucial for effective transcriptome studies is detailed knowledge of the cell biology of the interaction. This has been thoroughly documented for the flax rust fungus [27–31], thus allowing patterns of plant or pathogen gene expression to be placed in the context of structural and physiological processes that occur during the interaction in terms of both pathogenicity and defense. There is good evidence, for example, that the speed of the plant response greatly influences the outcome of the interaction, *i*.*e*., whether it is compatible or incompatible [32–36].

Flax rust has been a key model system for studies of biotrophic fungal pathogens for over 70 years. It was the pathosystem that Flor used in the development of the gene-for-gene hypothesis which proposes that an incompatible outcome of a plant-pathogen interaction is determined by recognition of a protein encoded by a pathogen *Avr* gene by a protein encoded by a plant resistance gene [37]. Multiple Avr proteins and effectors have been identified in flax rust [27, 38–40], which allows for comparative analyses of their expression as well as correlation between expression profiles and the cell biology of the interactions.

Here we describe an RNA-Seq analysis of *M*. *lini* transcriptomes during the establishment of disease in flax, with a focus on genes that encode predicted secreted proteins. The transcriptomes represent germinated urediniospores and infected flax seedlings collected from 2 to 8 days post inoculation (dpi). We investigate the expression profiles of known *Avr* and effector genes across several stages of infection. qPCR is used to complement the RNA-Seq data for selected genes. We find 58 previously uncharacterized *M*. *lini* genes encoding secreted proteins that cluster with six known *Avr* genes based on analysis of their expression patterns. In addition, we carry out a detailed analysis of the complement and transcription of CWDEs. The CWDE genes are preferentially expressed in germinated spores and during late stages of infection. The *M*. *lini* CWDE transcripts show multiple patterns of expression, apparently unrelated to their catalytic activity or potential substrates.

## Materials and methods

### Plant and rust material

Flax (*L*. *usitatissimum*, variety Hoshangabad) leaves were brush inoculated with air-dried dikaryotic urediniospores of *M*. *lini* strain CH5 and infection initiated as described by [41]. Plants were transferred to a greenhouse (23°C day and 13–15°C night temperatures) at 2 dpi. Uninoculated flax leaves were used as controls. Inoculated leaf samples were collected at 1–10 dpi for qPCR and at 2–6 dpi and 8 dpi for RNA-Seq experiments. Three biological replicates were included for each infection time point. For some samples, such as germinated urediniospores, a fourth replicate was included for the purposes of qPCR analysis and used in place of one of the samples used for RNA-Seq analysis. Urediniospores were collected from 13 dpi flax leaves. Germinated urediniospores were obtained after growth at 16°C on water overnight (for qPCR) or for 6h (for RNA-Seq). Flax rust haustoria were isolated from flax leaves 6 dpi by affinity chromatography as described by [27].

### Quantitation of flax leaf infection

Plant and flax rust samples were ground to a fine powder in liquid nitrogen in a mortar and pestle. A small amount of the powder was used for genomic DNA (gDNA) extraction using a DNeasy plant mini kit according to the manufacturer’s instructions (Qiagen, Hilden, Germany) or following the phenol-chloroform method as described by [42]. The concentration of gDNA was assessed by spectrophotometry and the relative amounts of flax and *M*. *lini* gDNA in each sample were determined using qPCR with primers specific for flax and *M*. *lini* glyceraldehyde-3-phosphate dehydrogenase (*GAPDH*). Sequence information for primers is listed in S1 Table. qPCR reactions were conducted using the Rotor-Gene Q Thermal Cycler (Qiagen), with 300 ng gDNA, 180 nM primers and QuantiFast SYBR Green Master Mix (Qiagen). Three or four technical replicates were included for each sample. The amplification was set as an initial step of 50°C for 1 min and polymerase activation for 5 min at 95°C followed by 35 cycles of 10 s at 95°C, 20 s at 58°C or 59°C and 30 s at 72°C. qPCR data were acquired after each annealing step and analyzed using the comparative quantification function of the Rotor-Gene Q series software (version 2.0.3; Qiagen), with the comparative concentration of samples at 5 dpi of the first biological replicate as the calibrator.

### RNA isolation

Total RNA was extracted using a Qiagen RNeasy plant mini kit, according to the manufacturer’s instructions. For RNA samples to be analyzed by qPCR, DNA was removed by incubation with 0.33 μg/μl RQ1 RNase-free DNase (Promega Corporation, Madison, WI, USA) at 37°C for 1 h followed by enzyme inactivation at 65°C for 10 min. For RNA samples used for sequencing, DNA was removed by a 40-min on-column digestion with a Qiagen RNase-free DNase kit based on the manufacturer’s instructions. The concentration of RNA in the samples was measured by spectrophotometry and its integrity checked by running 1 μg of the RNA sample on a 1.5% RNase-free agarose gel in TAE [1 mM ethylene diamine tetraacetic acid pH 8.0, 40 mM Tris, 19 mM acetic acid, 0.1% (v/v) diethyl pyrocarbonate (Sigma-Aldrich, St Louis, USA)].

### qPCR expression analysis

RNA was reverse transcribed using the SuperScript II reverse transcriptase protocol (Thermo Fisher Scientific Corporation, Waltham, Massachusetts, USA) for first strand cDNA synthesis with 1 μg of the oligonucleotide dT_12-18_ primer mixture, according to the manufacturer’s instructions. To protect RNA from degradation, 1 unit/μl of RNase inhibitor (RNasin or RNaseOUT; Promega Corporation, Wisconsin, USA) was used. For each sample, a negative control without the addition of reverse transcriptase was included to monitor any DNA contamination. The cDNA samples were diluted with RNase-free water, prior to use.

Of six *M*. *lini* genes tested, three were selected for normalization of qPCR assays. The *M*. *lini* gene encoding β-tubulin (TUB1; AF317682) had been reported previously. The other two *M*. *lini* genes were identified by searching the translated EST database of *M*. *lini* CH5 [27] with sequences for *M*. *larici-populina* GAPDH (EGF98632) and *Phaeosphaeria nodorum* hypothetical protein SNOG10408 (SNOG408; XP_001800679) using the CLC Genomic Workbench, version 6 (Qiagen). The *M*. *lini* sequences were aligned against *M*. *larici-populina* sequence data on the Joint Genome Institute and NCBI websites to verify intron and exon boundaries for the purposes of primer design. Primers designed for the three reference genes, namely *TUB1*, *GAPDH* and *SNOG408*, are listed in S1 Table.

Transcript levels of the three reference genes were determined in three biological replicates of samples of flax leaves 3–8 dpi with *M*. *lini*. Consistency in the transcription of *TUB1*, *GAPDH* and *SNOG408* genes across the infection time-course was assessed using geNorm (qbase+ version 3.0, Biogazelle, Zwijnaarde, Belgium) [43], Bestkeeper (version 1.0) [44] and NormFinder (version 0.953) [45]. The amplification efficiency and correlation coefficients (*R*^2^) of each pair of primers were calculated using the LinRegPCR program [46] and the results are shown in S2 Table. The use of multiple reference genes in qPCR has been shown to generate more reliable results than use of a single gene [43, 47]. Thus, the geometric mean of the Ct values for *TUB1*, *GAPDH* and *SNOG408* was used to normalize the expression values of the genes of interest [43, 48].

*Avr* gene expression was determined using the comparative quantification function of the Rotor-Gene Q software (version 2.0.3, Qiagen), with values in each sample expressed with respect to those in 5 dpi cDNA. The relative expression of *Avr* genes was then determined as a ratio to the geometric mean of the comparative concentration of the selected reference genes.

### RNA-Seq data collection and trimming

Next-generation sequencing was performed at the Australian Genomic Research Facility (Melbourne, VIC, Australia) after quality assessment using HiSeq Control software (v1.4.8) and Real Time Analysis (v1.12.4.2). cDNA libraries were generated from the RNA samples using the TruSeq protocol (version 2; Illumina, Inc., San Diego, CA, USA) as described by the manufacturer. The 24 cDNA libraries were sequenced using the Illumina consensus assessment of sequence and variation pipeline (version 1.8.2). Samples were grouped into sequencing lanes as listed in S3 Table and a total of 1.33 billion RNA-Seq single-end, non-strand-specific reads 50 bp in length were obtained. The raw reads were preprocessed using Condetri for adapter-sequence trimming [49] and Trimmomatic for quality-based sequence trimming [50]. The Trimmomatic criteria used were mostly default settings, *i*. *e*. Trimmomatic-0.32, ILLUMINACLIP: TruSeq3-SE. fa: 2:30:10, LEADING:3, TRAILING:3, SLIDINGWINDOW:4:15, MINLEN:36.

### Read mapping

The trimmed reads were aligned to the flax rust genome assembly v1 (genotype CH5) [51] using default settings in Bowtie v2.2.2 [52]. Reads that did not map to the flax rust genome were subsequently aligned to the flax genome v1 [53].

Only 0.9% of the reads from uninoculated plant samples aligned to the flax rust genome and only 0.15% of the reads from germinated spores that remained unaligned to the flax rust genome aligned to the flax genome. All such reads were eliminated from further analysis.

Reads that aligned to the flax rust genome were used in *Cufflinks* v2.2.0 [54] to generate a separate transcript assembly for each replicate for each of the samples that contained fungal material. These separate assemblies were then merged using *cuffmerge* followed by comparison with annotation v1 of the flax rust genome [51] using *cuffcompare*.

### Differential gene expression analysis

Raw read mapping values were converted to fragments per kilobase per million mapped reads (FPKM) and differential gene expression was analyzed using the *cuffdiff* module of *Cufflinks* [54, 55]. To search for genes with similar expression profiles to those of known flax rust effectors, transcripts for 1,085 secreted proteins predicted from the *M*. *lini* proteome [51] were used as the initial reference dataset. Transcripts with unique coding sequences that included a start codon and with a total FPKM value across the infection time series greater than 5 were retained for expression analysis.

Input data for heat-map presentation were converted into log base 2 (log_2_) FPKMs. These were averaged for biological replicates and *k*-means clustering with Euclidean distance was used to find genes that were co-expressed. Rather than absolute expression, we used the amount by which expression of each gene deviates in a specific sample from the average of the gene’s expression across all samples. Heat-maps were plotted with heatmap.2 in the R environment. Prediction of effector candidates from the predicted *M*. *lini* secretome was performed using EffectorP 2.0 [56].

### Identification of putative cell wall degradation enzymes

Genes encoding proteins with carbohydrate active enzyme (CAZyme) modules were identified from the predicted proteome described in [51] using the CAZyme annotation dbCAN2 meta server [57]. Three dbCAN2 bioinformatics tools (HMMER, DIAMOND and Hotpep) [48] were employed to identify putative CAZymes. The default cut-offs of these tools were: HMMER: E-value < 1e-15, coverage > 0.35; DIAMOND: E-value < 1e-102; Hotpep Frequency > 2.6, Hits > 6, as described on the dbCAN2 meta server. Proteins were only considered to be CAZymes if they were positive by at least two tools. CWDEs were identified by homology to CAZymes that have been previously characterized with activities that target cell wall components and this analysis relied on homology to characterized CAZymes found in the CAZY database [58] and NCBI reference protein database (refseq protein) and were predicted to be secreted. The presence of secretion signal peptides (SPs) for these CWDE candidates was predicted using SignalP4.1 (D-score > 0.45) [59] and SecretomeP2.0 (NN-score > 0.6) [60]. This meant that proteins with classical or non-classical SPs were included in the CWDE data set. To ensure that assembly errors did not result in exclusion of putative CWDEs, homologous proteins from three *Melampsora* species (JGI: *M*. *larici-populina*, *M*.*allii-populina* 12AY07 and *M*. *medusae* f. sp. *deltoidae* Mmd05TRE539) were examined for secretion signals. Furthermore, any putative *M*. *lini* CWDEs that had additional domains indicating that assembly errors had resulted in two genes annotated as one gene were excluded from transcriptome analysis. Detailed analysis of CWDEs excluded proteins predicted to specifically degrade fungal cell wall components.

## Results

### qPCR analysis of rust effector gene expression

**Fig 1:**
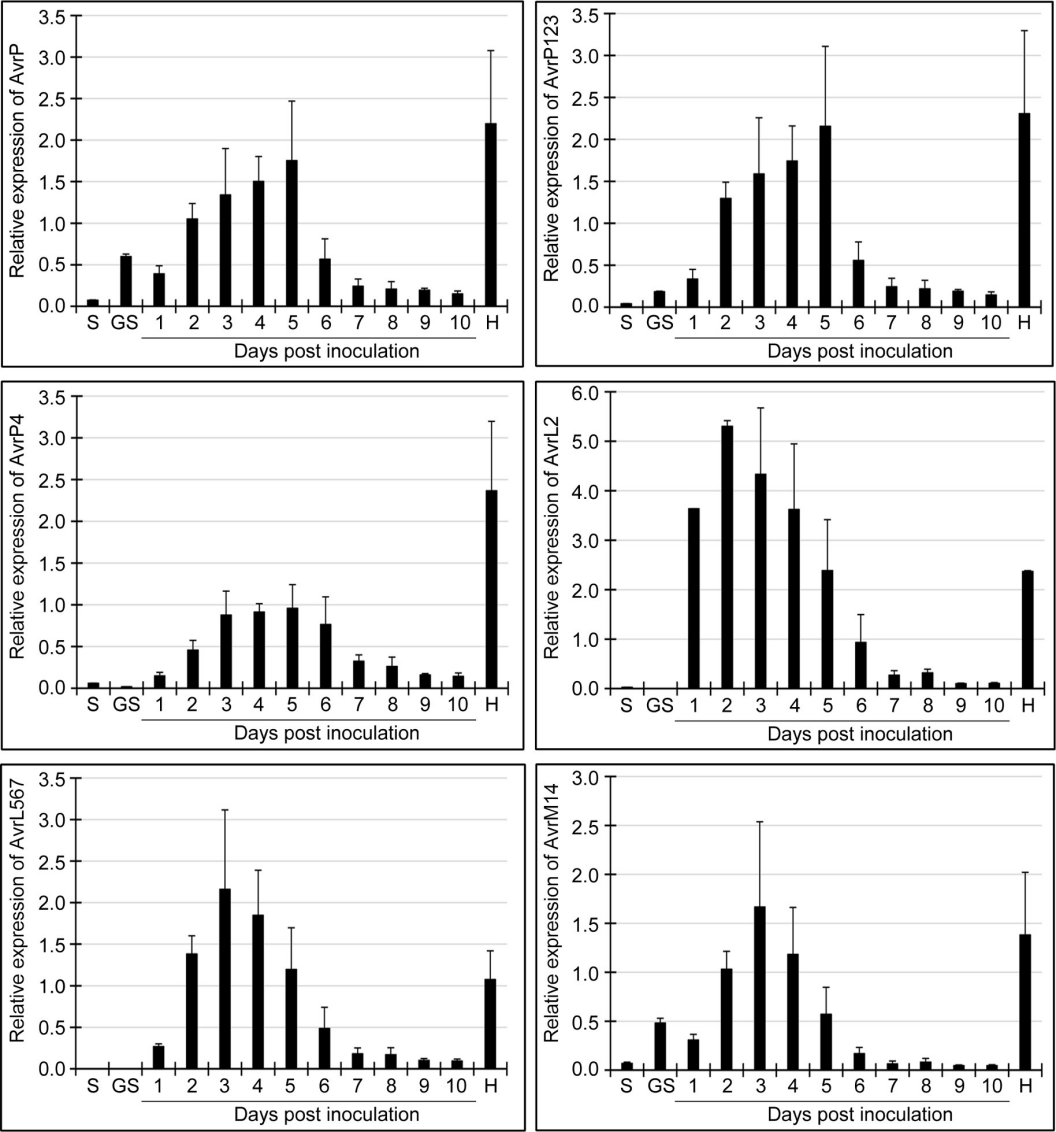
Transcript levels for *AvrP*, *AvrP123*, *AvrP4*, *AvrL567*, *AvrM14* and *AvrL2* genes determined using qPCR. Samples were obtained from resting spores (S), *in vitro* germinated spores (GS), infected flax leaves 1–10 dpi (days post inoculation) and isolated haustoria (H). Error bars show the standard error of the means of three biological replicates.

Transcript levels of six *M*. *lini Avr* genes, namely *AvrP*, *AvrP123*, *AvrP4*, *AvrL567*, *AvrM14* and *AvrL2*, in ungerminated spores, *in vitro* germinated spores, infected flax leaves 1–10 dpi and isolated haustoria were determined using qPCR. *AvrM* was excluded from this analysis because the nucleotide sequences of the virulence and avirulence alleles of *AvrM* were 99% identical and it was not possible to amplify *AvrM* alone. Transcript levels for all six *Avr* genes were low in ungerminated and germinated spores (Fig 1). In all cases, *Avr* gene expression increased after inoculation of the flax leaves, reaching a peak 2–5 dpi, before decreasing to low levels 7–10 dpi. Transcript levels for all six *Avr* genes were much higher in isolated haustoria than infected leaves at the same time point (6 dpi) (Fig 1), consistent with haustoria being the main location for *Avr* genes expression in infected leaves.

### RNA-Seq analysis of flax rust transcriptomes in spores and during plant infection

RNA-Seq data were obtained from three biological replicates of eight samples, including germinated rust spores, uninoculated flax leaves and flax leaves collected 2, 3, 4, 5, 6 and 8 dpi (S3 Table). Application of quality control criteria eliminated approximately 7% of the reads from further consideration. After trimming, the remaining 93% were mapped sequentially against the genome assemblies of *M*. *lini* and flax [51, 53].

**Fig 2:**
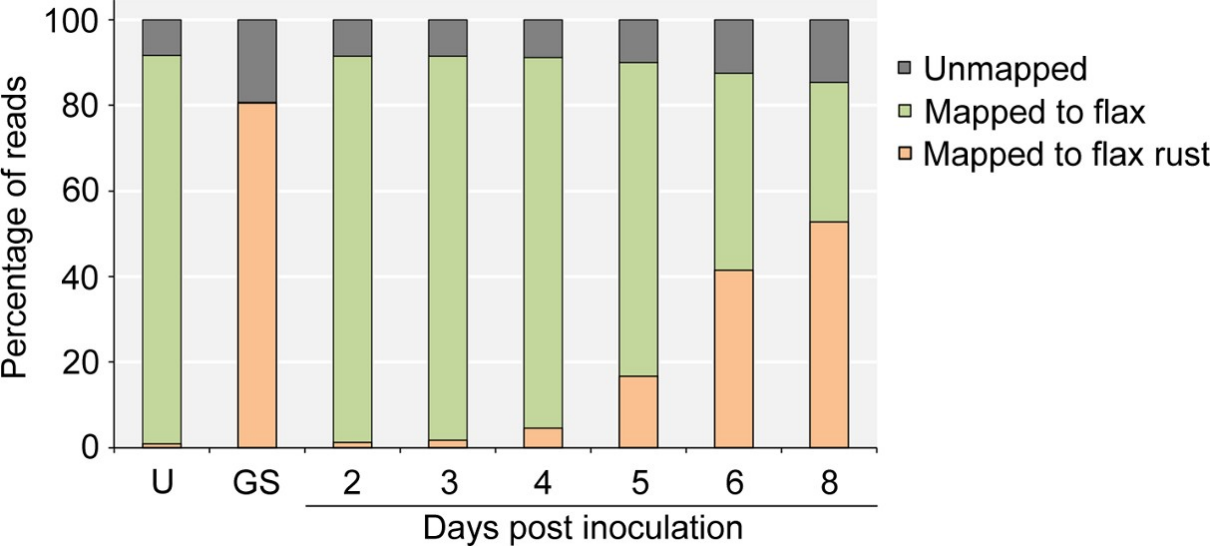
Mapping of the reads to the genomes of flax rust and flax. The percentages of reads that mapped to the flax rust and the flax genome are shown on the vertical axis.

The majority of reads from uninoculated flax leaf samples mapped to the flax genome and most reads from the germinated spore samples mapped to the genome of *M*. *lini* (Fig 2). In both cases, less than 1% of reads mapped to the genome of the species not included in the sample. This non-specific read mapping is likely to be due to alignment to highly conserved genes present in both species and these reads were eliminated from further analysis. In the infected flax leaf samples, the number of reads that mapped to the genome of *M*. *lini* increased from about 1% at 2 dpi to over 50% at 8 dpi, a trend consistent with the increasing pathogen load during the infection time-course (S1 Fig). An average of 8% of reads from uninoculated flax leaves, 15% of reads from inoculated leaves and 19% of reads from germinated spores failed to align to either the flax or flax rust genomes. Apart from possible contaminants and the different genotype of the flax cultivars used in this study (Hoshangabad) from the reference flax genome (CFC Bethune), it is likely that incompleteness of the two genome assemblies, in particular that of *M*. *lini*, contributed to the number of unmapped reads. The fraction of unmapped reads increased during the infection time-course as the fungal biomass increased (Fig 2).

Programs within the Cufflinks suite were used to assemble the reads that aligned to the genome of *M*. *lini* into a transcriptome database and comparison of the expression levels of the assembled transcripts showed that samples from different biological replicates were less varied than those collected from different post-inoculation time points (S2 Fig).

### *Avr* genes exhibit similar patterns of expression in the RNA-Seq assay

**Fig 3:**
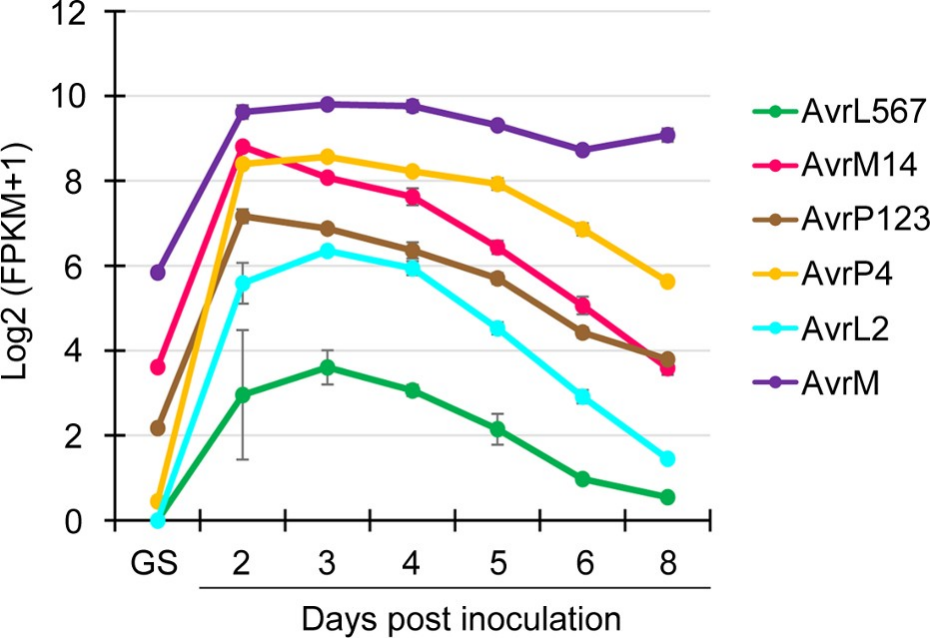
Transcript abundance of *M*. *lini Avr* genes in germinated spores and during infection. Transcript levels are shown on the vertical access as log base 2 (FPKM count + 1). Error bars show the standard error of the means of three biological replicates. GS: *in vitro* germinated spores.

Initial analysis of the RNA-Seq results focused on the expression patterns of known *M*. *lini Avr* genes. The data showed that transcript levels for the known *M*. *lini Avr* genes, namely *AvrL2*, *AvrL567*, *AvrM* (including *avrM*), *AvrM14*, *AvrP123* (including *AvrP*), and *AvrP4*, displayed similar expression profiles across the infection time-course (Fig 3). Transcripts of all the *Avr* genes were substantially more abundant in inoculated flax leaves than they were in germinated spores. During infection, transcript levels were highest at 2–3 dpi and subsequently decreased as infection proceeded. For *AvrL2*, *AvrL567* and *AvrM14*, the fall in transcript abundance was quite rapid, but for *AvrP123* (*AvrP*) and *AvrP4*, the fall was slower, and for *AvrM* (*avrM*), transcript levels fell only slightly during the ensuing 5 days.

We also performed a cluster analysis of the expression profiles of 623 *M*. *lini* genes predicted to encode secreted proteins [51]. These genes were divided into five groups based on their patterns of expression (S3 Fig, S4 Table). The genes within each group had no obvious similarities in functional annotation [51]. What was especially notable, however, was that the known *M*. *lini Avr* genes listed above occurred within a single group (Cluster 3 in S3 Fig). This cluster contained 58 other genes that had a similar expression profile, with lowest expression in germinated spores and highest expression during early time points of infection (S3 Fig). If the timing of gene expression is related to protein function and plant recognition, then it is possible that some of these other 58 genes also encode effectors with the potential to function as Avr proteins.

**Fig 4:**
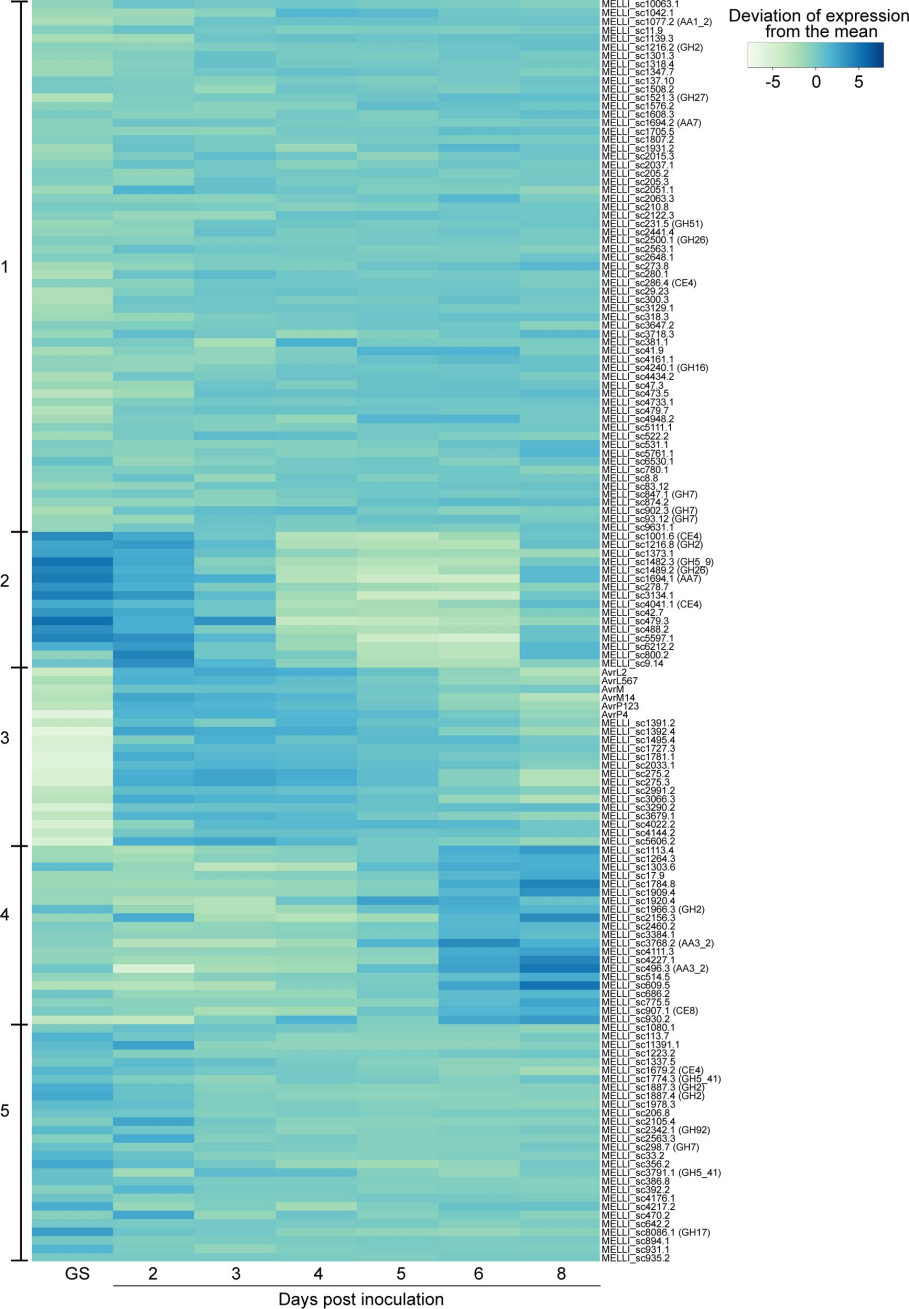
Clustering of *Avr* genes, candidate effectors and CWDEs based on expression profiles. Expression values of transcripts were converted into log base 2 of FPKM counts prior to use. Genes with similar patterns of expression are clustered into five groups using the *k*-means algorithm. The elbow plot method was used to estimate the expected number of clusters in the data [61]. Genes within a cluster are listed in alpha-numerical order by gene designation. The genes included are a subset of those in the full heatmap of the secretome shown in S3 Fig. GS: *in vitro* germinated spores.

Excluding *Avr* genes, 112 of the remaining 617 secreted-protein genes shown in S3 Fig and listed in S4 Table were predicted to be effectors using EffectorP 2.0 [56], with 53 in Cluster 1 (18.3%), 10 in Cluster 2 (21.3%), 15 in Cluster 3 (25.9%), 16 in Cluster 4 (27.1%) and 18 in Cluster 5 (11.0%) (Fig 4). The 15 genes that grouped with the *Avr* genes in Cluster 3 included two paralogs of *AvrL2* (MELLI_sc275.2 and MELLI_sc275.3) and the virulence allele of *AvrL567* (MELLI_sc1392.4).

### Prediction and expression analysis of CWDEs

During plant colonization, plant pathogens secrete numerous CWDEs to weaken and break through the barriers formed by plant cell walls. This process is essential for facilitating plant invasion by plant fungal pathogens. To determine the expression features of *M*. *lini* CWDEs during infection, putative CWDE genes in the *M*. *lini* genome were identified and their expression profiles determined using the RNA-Seq data.

CAZyme modules are one of the most typical characteristics for identifying proteins with potential capacity to degrade plant cell walls. Based on sequence or functional features of proteins in carbohydrate-active enzyme families, CAZyme modules in CWDEs are assigned to six main families, namely glycoside hydrolases (GHs), glycosyltransferases (GTs), polysaccharide lyases (PLs), carbohydrate esterases (CEs), auxiliary activities (AAs) and carbohydrate-binding modules (CBMs) [58]. Analysis of the flax rust proteome using dbCAN2 showed that of the putative 26,443 proteins predicted by [51], 175 had predictable CAZyme motifs. Based on the selection criteria described above, 13 genes with assembly errors, 95 genes that produce proteins with no homology to characterized CWDEs acting on plant cell wall components and nine genes with a total FPKM across the infection time-course of less than 5 were excluded from further consideration. Moreover, three pairs of CWDE genes were found to share high homology (over 99.8% identity) so only one from each pair was retained in the expression analysis. The remaining 55 CWDE candidate proteins were classified into four AA, two CE, 15 GH and three PL families based on the CAZyme modules they contained (S5 Table).

**Fig 5:**
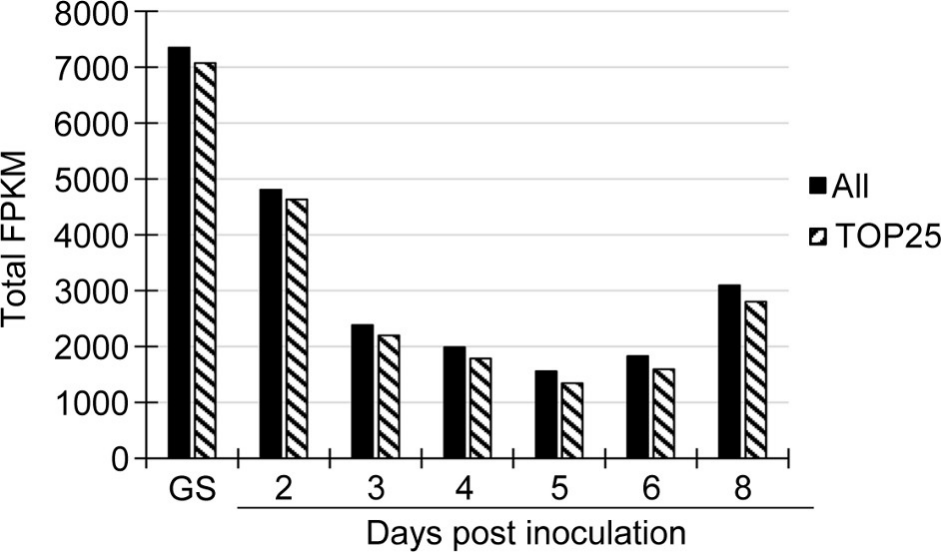
Comparison of expression of the 25 most highly expressed (TOP25) and all (All) CWDE genes. Expression profiles of CWDE genes in the predicted *M*. *lini* transcriptome were shown in terms of total FPKM at each time point of infection. GS: *in vitro* germinated spores.

Global expression patterns for the *M*. *lini* CWDE transcripts were evaluated by summing the FPKM values of all of these genes at each infection time point (Fig 5). In terms of total transcript abundance at each time point, the highest level of gene expression occurred in germinated spores in which the total FPKM was 1.5 to 4.7-fold higher than that at any of the infection time points. In infected plant tissues, global transcript levels of the 55 CWDE genes were highest at 2 dpi, decreased over the ensuing 5 days before increasing between 7 dpi and 8 dpi (Fig 5). The total FPKM value of the 55 CWDE transcripts was generally low during infection. Only 25 genes had an FPKM value greater than 50 at one or more time points and a total FPKM during the infection time-course greater than 100. The total FPKM values at each time point of these 25 genes constituted 86% to 96% of that of all 55 CWDE transcripts (Fig 5), indicating that the contribution of the remaining 30 genes to the transcriptome was minimal. Of these 25 genes, 19 had a seven-fold or more change in expression at different time points and were considered to be differentially expressed (Table 1).

**Table 1:** Expression analysis of the top 25 most highly expressed CWDE transcripts in the *M*. *lini transcriptome*.

Rank (total FPKM)	CAZyme family	Transcript	FPKM								Fold change	Rank (fold change)	Putative functions	Category of putative substrate
			GS	2 dpi	3 dpi	4 dpi	5 dpi	6 dpi	8 dpi	Total				
1	CE4	MELLI_sc1679.2	1021	676	441	446	327	213	100	3225	10.11	15	Acetyl-xylan esterase or chitin deacetylase	Hemicellulose, Chitin
2	GH5_41	MELLI_sc3791.1	432	72	509	476	168	103	242	2001	6.99	19	Putative mannanase	NA
3	AA3_2	MELLI_sc496.3	52	0	9	11	82	271	1239	1664	1240.08	1	Glucose-methanol-choline oxidoreductase	Lignin-related compounds, Cellulose
4	GH17	MELLI_sc8086.1	954	183	157	75	61	53	79	1563	17.64	13	β-1,3-glucosidase	Callose
5	PL14	MELLI_sc1172.2	139	1114	1	93	0	0	11	1358	969.32	2	β-1,4-glucuronan lyase	D-glucuronan
6	AA5_1	MELLI_sc3156.1	701	469	44	34	1	1	43	1293	415.91	4	Glyoxal oxidase (galactose oxidase, alcohol oxidase)	Lignin-related compounds
7	CE4	MELLI_sc1001.6	810	304	66	8	5	16	77	1287	132.63	8	Acetyl-xylan esterase or chitin deacetylase	Hemicellulose, Chitin
8	GH43_6	MELLI_sc2334.5	52	223	368	246	151	79	40	1159	8.96	17	α-1,5-L-arabinase	Hemicellulose, Pectins, Glycoproteins
9	CE8	MELLI_sc907.1	104	64	24	31	150	331	379	1083	15.39	14	Pectin methyl esterase	Pectins
10	GH16	MELLI_sc233.6	261	640	43	2	4	5	7	962	215.72	5	Endo-β-1,3–1,4-glucanase	β-1,3:1,4-glucans
11	AA7	MELLI_sc1694.1	537	102	84	2	0	0	46	772	499.27	3	Glucooligosaccharide oxidase	Cellulose
12	GH92	MELLI_sc2342.1	232	131	91	51	65	82	96	749	4.49	20	α-1,2-mannosidase	Glycoproteins
13	GH26	MELLI_sc2500.1	87	74	94	100	113	121	109	698	1.64	25	β-1,4-mannase or β-1,3-xylanase	Hemicellulose
14	GH3	MELLI_sc990.7	392	122	37	16	12	16	35	631	29.31	12	Glucan β-1,3-glucosidase or β-glucosidase	Callose, Cellulose
15	GH5	MELLI_sc1379.2	155	65	51	58	54	56	71	510	3.02	23	Putative β-1,3-glucanase	Callose
16	GH26	MELLI_sc1489.2	319	65	14	2	1	3	37	440	201.72	7	β-1,4-mannase or β-1,3-xylanase	Hemicellulose
17	GH16	MELLI_sc765.2	114	46	42	29	32	36	40	339	3.89	22	Endo-β-galactosidase	Glycoproteins
18	AA3	MELLI_sc278.8	23	39	44	51	53	48	37	296	2.21	24	Glucose-methanol-choline oxidoreductase	Lignin-related compounds, Cellulose
19	GH5_9	MELLI_sc1482.3	241	34	4	1	0	0	7	286	215.70	6	β-1,3-glucanase	Callose
20	GH16	MELLI_sc233.5	141	61	12	3	1	1	6	224	82.66	10	Endo-β-1,3–1,4-glucanase	β-1,3:1,4-glucans
21	GH2	MELLI_sc1887.3	99	39	15	16	13	15	15	213	7.15	18	Putative β-mannosidase, β-galactosidase or β-glucuronidase	Hemicellulose, Pectins, Glycoproteins
22	GH2	MELLI_sc1887.4	99	30	15	11	10	10	12	187	9.20	16	Putative β-mannosidase, β-galactosidase or β-glucuronidase	Hemicellulose, Pectins, Glycoproteins
23	PL1_4	MELLI_sc3487.3	51	12	17	19	16	37	31	184	4.00	21	Pectin lyase	Pectins
24	GH2	MELLI_sc1216.8	54	70	18	1	1	0	14	158	57.67	11	Putative β-mannosidase, β-galactosidase or β-glucuronidase	Hemicellulose, Pectins, Glycoproteins
25	AA3_2	MELLI_sc3768.2	4	0	0	1	20	91	25	141	92.19	9	Glucose-methanol-choline oxidoreductase	Lignin-related compounds, Cellulose

Transcript abundance is shown in terms of FPKM values, with the highest FPKM in red and the lowest in yellow.

The cluster analysis of 623 secreted protein genes included 28 of the 55 CWDEs, including 15 of the 25 most highly expressed CWDEs. In contrast to the conserved patterns of expression observed for the *M*. *lini Avr* genes, the patterns of *M*. *lini* CWDE gene expression, like those for the predicted *M*. *lini* effector genes, fell into multiple clusters, but were absent from Cluster 3, which contained the *Avr* transcripts (Fig 4). CWDEs that contained CAZyme motifs from the same family, had similar catalytic activities or targeted the same substrate class (S5 Table) were not necessarily clustered together.

To better understand the expression properties of CWDE genes in the *M*. *lini* transcriptome, the expression profiles of the 25 most highly expressed CWDE candidates were further assessed according to their predicted catalytic function(s) and the cell wall component(s) putatively targeted by their encoded enzymes, namely cellulose, hemicellulose, pectin, glycoproteins, lignin and the related compounds, callose and chitin (Table 1). Two CWDEs from the GH26 CAZyme family were likely to act specifically on hemicellulose, and another four CWDEs from two different CAZyme families (GH2 and GH43) might also target pectin or glycoproteins as well as hemicellulose. Two proteins from the CE4 family may also target hemicellulose but could also act on chitin. The transcript abundance of six of these eight genes was enriched in germinated spores or at 2 dpi and then rapidly declined as infection continued. Exceptions were the putative α-1,5-L-arabinase from GH43 (MELLI_sc2334.5) whose expression peaked at 3 dpi and decreased thereafter, and the GH26 CWDE (MELLI_sc2500.1) that showed constitutively low levels of expression throughout the infection time-course.

A cohort of four CWDEs (MELLI_sc990.7, MELLI_sc1379.2, MELLI_sc1482.3 and MELLI_sc8086.1) was predicted to encode β-1,3-glucanases; although one of these (MELLI_sc990.7) may produce a β-glucosidase that degrades cellulose. All but one of these genes were differentially expressed, with abundant expression in germinated spores. The exception was MELLI_sc1379.2 whose expression was relatively low and steady during infection, and the expression levels between germinated spores and infected leaf samples were less varied than the other three genes in the cohort. The accumulation of transcripts in germinated spores or early infection was also detected for CWDEs that might have specific catalytic activities on glycoproteins (MELLI_sc765.2 and MELLI_sc2342.1), β-1,3:1,4-glucans (MELLI_sc233.5 and MELLI_sc233.6) and D-glucuronan (MELLI_sc1172.2).

Changes in expression of CWDEs that had potential to act on pectin were relatively gradual and limited. The expression of the CE8 CWDE gene predicted to produce pectin methyl esterase (MELLI_sc907.1) gradually increased as infection proceeded, while expression of the putative pectin lyase encoded by the PL1 gene (MELLI_sc3487.3) remained low throughout the infection time-course.

Five genes from three AA families (AA3, AA5 and AA7) had predicted enzymatic activities on lignin-related compounds, cellulose or both. Among these genes, MELLI_sc1694.4 from AA7 and MELLI_sc3156.1 from AA5 had the potential to interact specifically with cellulose and lignin-related compounds, respectively. Both of these genes were most highly expressed in germinated spores. However, their expression in infected leaf samples remained low, apart from a high level of gene expression detected for MELLI_sc3156.1 at 2 dpi. The three genes from the AA3 family that were likely to encode proteins that interact with both lignin-related compounds and cellulose were either expressed constitutively at low levels (MELLI_sc278.8) or were most highly expressed during late infection stages (MELLI_sc3768.2 and MELLI_sc496.3). This was especially true for MELLI_sc496.3 for which transcript levels at 8 dpi were much higher than at earlier time points. The FPKM value at 8 dpi was 1240-fold higher than that at 2 dpi in infected leaves and about 24-fold higher than that in germinated spores.

## Discussion

With the rapid development of genomics and transcriptomics, and their increasing application in the field of plant-pathogen interactions, high-throughput sequencing has become an indispensable technology that assists novel gene identification and prediction of protein function as well as characterization of the role of proteins in disease development [62–67]. The present study used a sequencing-based approach to explore expression during plant infection of two categories of *M*. *lini* secreted proteins, namely Avrs and CWDEs. The results reveal striking similarities in the expression patterns of known *M*. *lini Avr* genes and identify a set of co-expressed secreted proteins that are strong effector candidates. Transcript abundance for the majority of *M*. *lini* CWDEs was generally low but for those CWDEs that were strongly expressed, transcript levels tended to be highest in germinated spores and during early infection.

### *M*. *lini Avr* genes have similar patterns of expression

A striking outcome from the analysis of the patterns of *M*. *lini* gene expression is that the six known *Avr* genes, *AvrM* (*avrM*), *AvrM14*, *AvrL2*, *AvrL567*, *AvrP123* (*AvrP*) and *AvrP4*, all occur in the same cluster (S3 Fig, Cluster 3), with similar patterns of early expression during infection and very low expression in germinating spores (Figs 1 and 4). This cluster of 64 secreted protein genes also includes two paralogs of *AvrL2*, the virulence allele of *AvrL567* and 12 predicted effectors. For *Avr* genes, transcript abundance was also high in haustoria (Fig 1). One possible explanation for this common early-expression pattern for the *M*. *lini Avr* genes is that effective resistance by the host may require that R-protein mediated recognition occurs early during the infection process. Once infection is well established, host defense may already be compromised to the extent that recognition of later expressed effectors would be unable to trigger resistance. Hence *Avr* genes would be over-represented among effectors expressed early during infection. The observed *Avr* gene co-expression in *M*. *lini* suggests that expression profiling may provide a valuable approach to identify *Avr* genes in other rust pathogens. Indeed, expression profiling of rust secretomes has identified clusters with similar expression patterns in several pathogen species that are closely related to *M*. *lini* [68]. In the case of *Puccinia graminis* f. sp. *tritici* (*Pgt*), this cluster included the only other known rust *Avr* genes, *AvrSr50* and *AvrSr35* [69, 70]. While in *M*. *larici-populina*, genes showing similar expression patterns are enriched in homologoues of *M*.*lini* avirulence factors [71]. Analogous clusters detected in *P*. *coronata* f. sp. *avenae*, *P*. *striiformis* f. sp. *tritici* and *Ustilago maydis* also showed enrichment for predicted effector genes, although to date no *Avr* genes have been identified in these species [72–74]. Interestingly, the *M*. *lini*-like patterns of *Avr* gene expression seem not to be applicable to all fungal pathogens. For example, five *Avr* genes identified in *Leptosphaeria maculans*, *AvrLm1*, *AvrLm2*, *AvrLm4-7*, *AvrLm11* and *AvrJ1*, all show peak expression around 7 dpi in canola, which is later than that for *M*. *lini Avr* genes [33].

This cluster analysis of *M*. *lini* secreted-protein gene expression (S3 Fig) and predicted-effector gene expression in particular (Fig 4), suggests several different patterns of effector gene expression during infection of flax by *M*. *lini*. However, apart from the clustering together of *Avr* genes, effectors with identical functional activities are not always closely clustered. Diversity in expression of effector genes has also been detected in various pathogenic oomycetes and fungi. For example, the RXLR effector genes in *Phytophthora cactorum*, the CRN effector genes in *P*. *capsica* and candidate effector genes from *Sclerotinia sclerotiorum*, *Zymoseptoria tritici* and *Verticillium nonalfalfae* all show a variety of expression patterns during plant infection [75–79]. Differences between patterns of effector gene expression likely reflect differences in modes of pathogen attack and this may in turn determine which effectors are more likely to be recognized by host resistance proteins, leading to different patterns of *Avr* gene expression in different pathosystems. The pattern of *M*. *lini Avr* gene expression exemplified by Cluster 3 may therefore only be diagnostic for *Avr* genes in the rust fungi.

### A limited number of *M*. *lini* CWDEs control degradation of plant cell wall components with distinct expression phases before haustoria formation and during sporulation

CWDEs are important for pathogenicity by disrupting the integrity of plant cell walls during the establishment of disease. From the predicted proteome of *M*. *lini*, a total of 55 genes were identified in the current study with potential to encode CWDEs, based on their sequence similarities to homologous enzymes with known functions. As shown in S5 Table, *M*. *lini* CWDEs were mainly constitutively expressed at low levels during infection. Of the 55 CWDE candidates identified, less than half (45%) showed relatively high levels of expression. However, these transcripts accounted for 86% to 96% of the total FPKM for all CWDE genes at each time point. This is in line with the infection feature observed for numerous biotrophic pathogens, namely, minimal quantities of enzymes are required to enable pathogen penetration without causing substantial damage to host plant cells [80].

In contrast to the expression profiling shown for the *M*. *lini Avr* genes, levels of the *M*. *lini* CWDE transcripts were generally high in germinated spores and during early (*e*.*g*. 2 dpi) infection. For some transcripts, gene expression was also up-regulated in late (*e*.*g*. 8 dpi) stages of infection. These expression patterns of CWDEs are consistent with the expected peak periods of plant cell wall degradation during infection, namely during early colonization of the host plant and during sporulation (6–10 dpi) when rust pustules erupt through the leaf surface [81]. High expression of the majority of CWDEs is also seen in aeciospores and urediniospores of *Cronartium ribicola*, the white pine blister rust [23] and during the early infection of wheat by *Pgt* [82]. While there is variation in the temporal expression of CWDE genes during these host-pathogen interactions and flax rust infection, a number of genes that are expressed in spores or during early infection encode CWDEs with callose degrading activity.

Pectin is one of the main components of the plant cell wall and is known to be an important target for pathogens during initial stages of infection. Removal of the methyl group from homogalacturonan by pectin methyl esterases (PMEs) is thought to potentially improve cleavage of the α-1,4-linkage of polygalacturonic acids. Degradation of pectin results in the loosening of wall structures and the exposure of other polysaccharides within the plant cell wall, such as cellulose and glycoproteins, to the action of CWDEs [83]. Therefore, genes coding for pectic enzymes, especially PMEs, are expected to be active during early infection, as observed in *P*. *parasitica* and *P*. *sojae* [84, 85]. However, during the infection of flax leaves by *M*. *lini*, the gene predicted to produce PME (MELLI_sc907.1) was strongly up-regulated during late infection, with more than 5-fold increases in expression compared to early infection. *C*. *ribicola* PME genes were also expressed in spores and infected pine stems [23] and during the infection of wheat by *Pgt* [82]. This could possibly be related to the disruption of plant tissues during sporulation. As pectin plays an essential role in cell-cell adhesion [86], the eruption of rust pustules through the leaf surface may require loosening of the junctions between cells by PME, consistent with the high expression of this gene late in infection. Based on sequence similarities to identified CWDEs, several CWDEs of *M*. *lini* that might interact with pectin were also found to potentially degrade hemicellulose and glycoproteins. These genes were often induced early in infection, although their overall transcript abundance was low over the infection time-course. In this case, further characterization of the biochemical activity of these enzymes is needed. In addition, the low expression of PME transcripts during early infection might be correlated with changes in plant cell wall polymers upon pathogen invasion. As reported in [87], flax seedlings secrete increasing amounts of PMEs during the first 24 hpi by *Fusarium oxysporum* to rapidly loosen the structure of pectin and release oligogalacturonides that can activate the plant immune response. In this situation, the pathogen may be able to penetrate between plant cells without employing large amounts of PMEs, as indicated by the low levels of PME gene transcription seen in *M*. *lini*. Furthermore, the expression patterns of PMEs during infection might also reflect the diversity in infection strategies between biotrophic, hemibiotrophic and necrotrophic pathogens.

Contrasting patterns of gene expression in different pathogen species have also been detected for genes encoding proteins with potential to degrade β-1,3-glucans and glycoproteins. As has been observed for *P*. *parasitica*, for example, the degradation of β-1,3-glucans and glycoproteins often occurs during middle and late phases of infection [84]. However, the three β-1,3-glucanase-encoding genes and the two genes coding for proteins with degradative activity towards glycoproteins of *M*. *lini* were most highly expressed in germinated spores. Apart from the wall degradation during initial stages of infection, early induction of β-1,3-glucanases and those proteins acting on glycoproteins in *M*. *lini* is likely to be correlated with extensive pathogen wall remodeling before formation of haustoria.

Altogether, our finding of conserved expression patterns for *M*. *lini Avr* genes during infection may provide a valuable approach to rapidly identify *Avr* genes among other rust fungi. Additional work is required to understand and leverage the host and pathogen factors that control initiation of *Avr* and CWDE gene expression. Such knowledge is expected to shed light on the development of novel and durable strategies for crop disease resistance.

## Supporting information

S1 Fig Pathogen load in infected flax leaves from 1 to 10 days post inoculation. Error bars indicate the standard error of the mean of three biological replicates.
(TIF)

S2 Fig Euclidian distances of flax rust transcripts from different samples and biological replicates. The Euclidian distances are scaled as shown at the top of the dendrogram. The tree was generated using CummeRbund. U: uninoculated flax leaves; GS: *in vitro* germinated spores; dpi: days post inoculation; bio rep: biological replicate.
(TIF)

S3 Fig Clustering of the *M*. *lini* secretome based on expression profiles. Expression values of transcripts were converted into log base 2 of FPKM counts prior to use. Genes with similar patterns of expression are clustered into five groups using the *k*-means algorithm. The cluster containing *Avr* genes is highlighted in the red box. Genes within a cluster are listed in alpha-numerical order by gene designation. GS: *in vitro* germinated spores.
(TIF)

S1 Table Sequence information for primers used in the qPCR and RNA-Seq experiments. (DOCX)

S2 Table Amplicon information for candidate reference genes. (DOCX)

S3 Table RNA-Seq lane allocations and resulting read data for 24 cDNA libraries (three biological replicates of eight samples) of flax (*L*. *usitatissimum*, cultivar Hoshangabad) leaves uninoculated or inoculated with flax rust (*M*. *lini*, strain CH5). (DOCX)

S4 Table Expression of genes encoding secreted proteins, cell wall degrading enzymes (CWDE) and known Avr proteins, and qPCR reference genes. Secretome cluster group is shown along with total expression and fold change between maximum and minimum expression values. Putative effectors predicted by EffectorP 2.0 are indicated.
(XLSX)

S5 Table Identification of candidate CAZymes in the flax rust genome using dbCAN2. Proteins encoded by genes listed in the table were checked by homology with proteins with characterized functions found in the CAZy and NCBI Reference Protein databases. Potential secretion signals were determined using SignalP4.1 (D score) and SecretomeP2.0 (NN-score). Y-SP: presence of classical signal peptides detected in the flax rust CWDE; Y-SP* classical secretion signal detected by SecretomeP2.0 Server and/or by comparative sequence analysis of proteins from other *Melampsora* species; Y-NC: non-classical secretion predicted for flax rust proteins by SecretomeP2.0; Y-NC* non-classical secretion predicted by SecretomeP 2.0 Server and/or by comparative sequence analysis of other *Melampsora* species proteins. The number and type of dbCAN2 tools (HMMER, DIAMOND or Hotpep Frequency and Hits) [57] that identified positive CAZyme motifs in each predicted protein are shown along with their E-values and Hotpep results. Predicted proteins containing two or more motifs for unrelated proteins that arose through assembly errors predicted transcripts missing from the predicted transcriptome are indicated and these were not used in expression analysis. Total expression in FPKM is also shown.
(XLSX)

S6 Table Expression of the *M*. *lini* transcriptome in germinated spores and during infection from 2 to 8 dpi. (XLSX)

S1 File References for S5 Table. (DOCX)
