# Supplementary figures and images for: Flax rust infection transcriptomics reveals a transcriptional profile that may be indicative for rust Avr genes
Source: PLoS One. 2019 Dec 12;14(12):e0226106. doi: 10.1371/journal.pone.0226106 (PMC6907798; doi:10.1371/journal.pone.0226106)

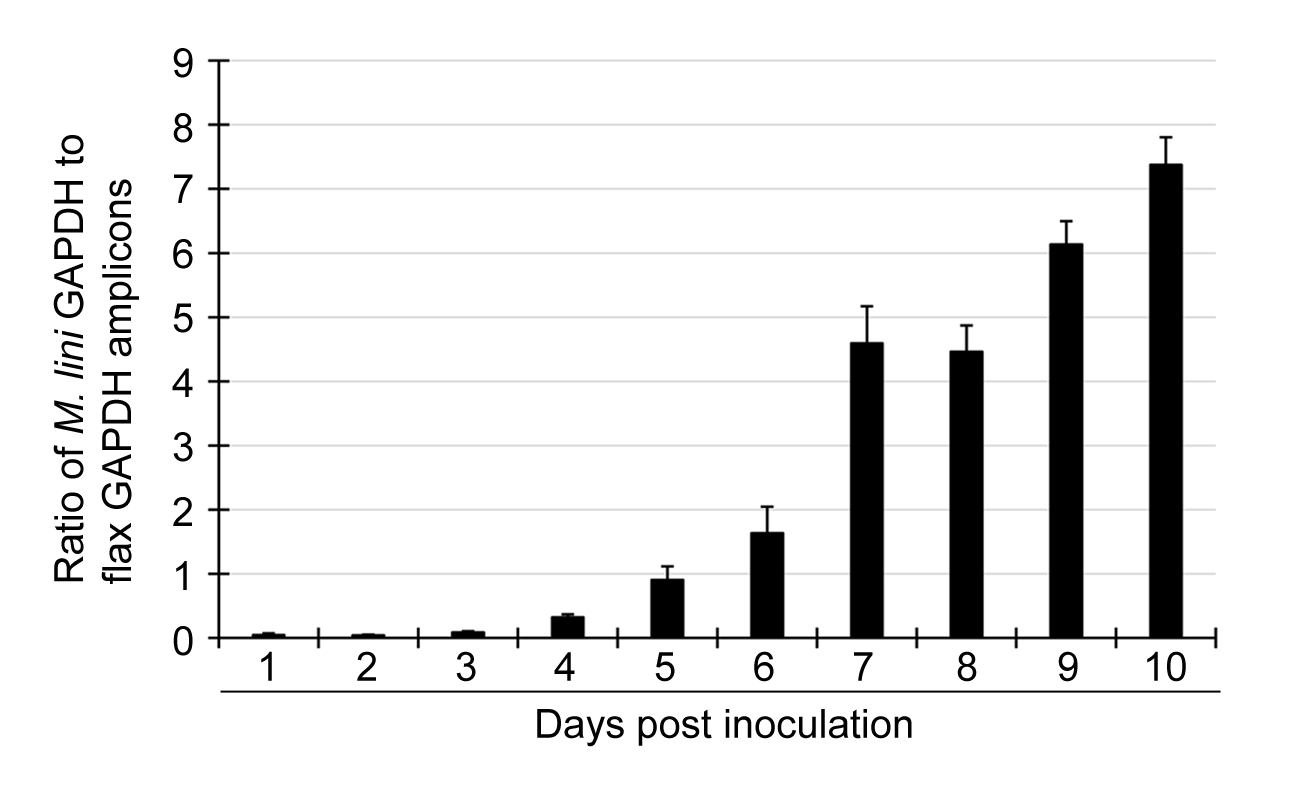

Supplement: S1 Fig — Error bars indicate the standard error of the mean of three biological replicates. (TIF) [file pone.0226106.s001.tif]

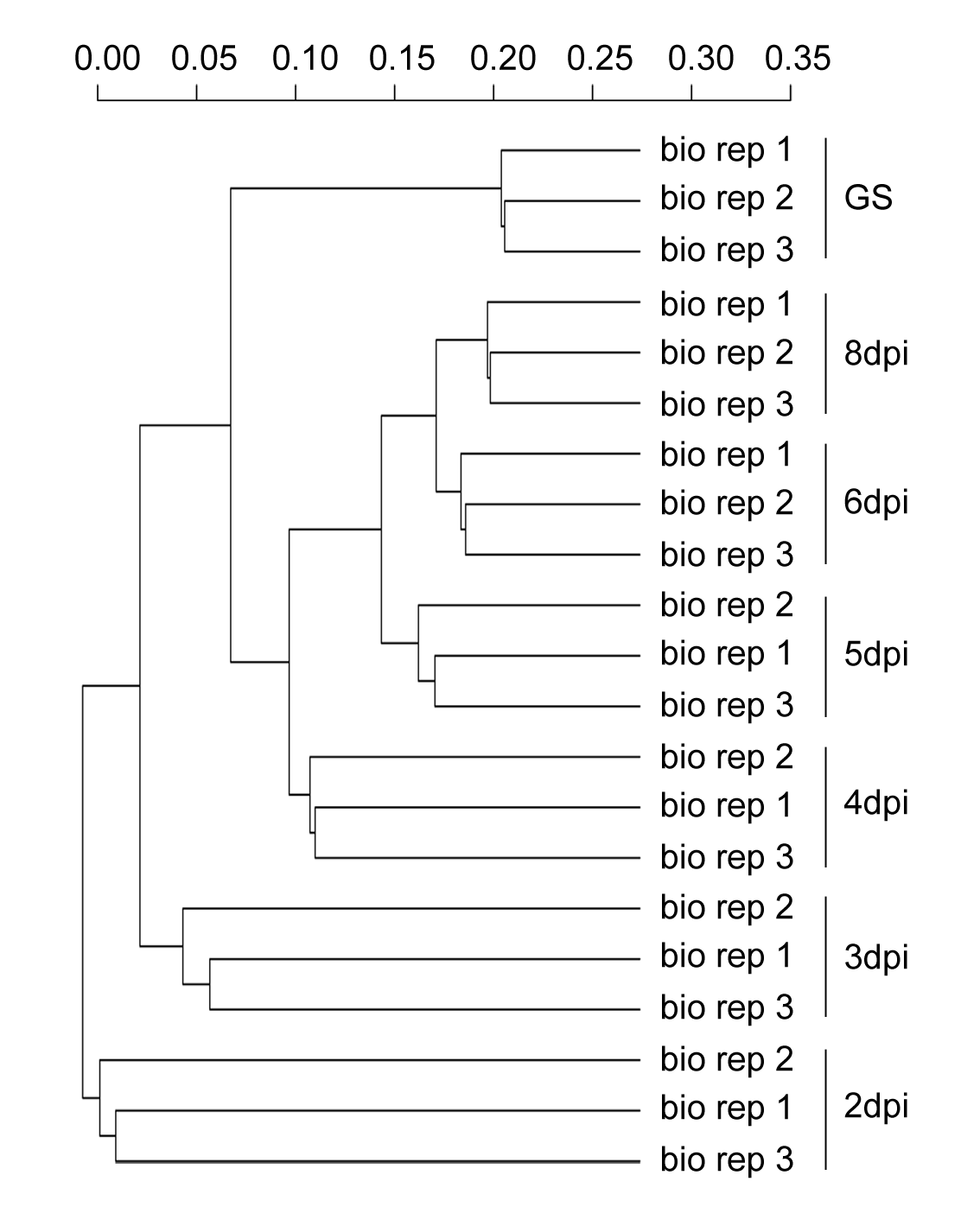

Supplement: S2 Fig — The Euclidian distances are scaled as shown at the top of the dendrogram. The tree was generated using CummeRbund. U: uninoculated flax leaves; GS: in vitro germinated spores; dpi: days post inoculation; bio rep: biological replicate. (TIF) [file pone.0226106.s002.tif]

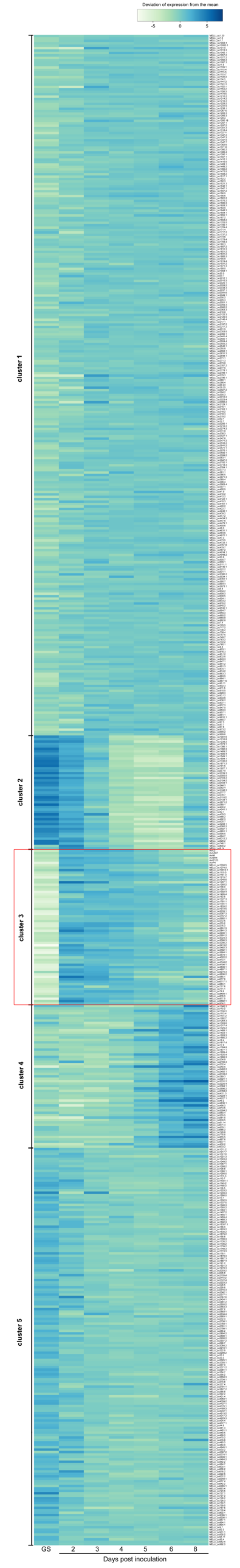

Supplement: S3 Fig — Expression values of transcripts were converted into log base 2 of FPKM counts prior to use. Genes with similar patterns of expression are clustered into five groups using the k-means algorithm. The cluster containing Avr genes is highlighted in the red box. Genes within a cluster are listed in alpha-numerical order by gene designation. GS: in vitro germinated spores. (TIF) [file pone.0226106.s003.tif]
