# Supplementary material for: Flax rust infection transcriptomics reveals a transcriptional profile that may be indicative for rust Avr genes
Source: PLoS One. 2019 Dec 12;14(12):e0226106. doi: 10.1371/journal.pone.0226106 (PMC6907798; doi:10.1371/journal.pone.0226106)
Supplement: S1 Table — (DOCX) [file pone.0226106.s004.docx]

**S1 Table.** **Sequence information for primers used in the qPCR and RNA-Seq experiments.**

| Gene |  | Primer Sequence [5’-3’] |
| --- | --- | --- |
| *GAPDH* (*M. lini*) | Forward | TGGTGCCGATTATGTTGTC |
|  | Reverse | AGGGCAGGGATGACTTTG |
| *GAPDH* (flax) | Forward | CAAACAAGGACTGGAGAGGTGGAAG |
|  | Reverse | GTGCTGCTGGGAATGATG |
| *Tubulin* | Forward | GCCAAAGGTCATTACACGG |
|  | Reverse | GAGAGTTCCCATTCCAGCAC |
| *SNOG408* | Forward | GAACCTCCAATGAATCCAAC |
|  | Reverse | TCTACCTGCCACATCCAAC |
| *AvrP* | Forward | AATCGCTCCTCTGACTCAAC |
|  | Reverse | AACCTCCGTACTCCACACTG |
| *AvrP123* | Forward | GGAGAGATTGAATGTATGTGCG |
|  | Reverse | CTTGATGGACCCCGAAAC |
| *AvrP4* | Forward | CGATAGGCAAGAAAAACAGGC |
|  | Reverse | TCCAAAAGGCAGTCACCAC |
| *AvrL567* | Forward | TACCAGCAGAGTTGACCAGAG |
|  | Reverse | GTCGCACCACTATTTCTTTGTG |
| *AvrM14* | Forward | GCCCTCTTCTTTGTCACTATCAG |
|  | Reverse | CCGCTTTTCTTCACCATTC |
| *AvrL2* | Forward | CGAGATCTTTACCATCATGG |
|  | Reverse | TAATGAATTAGGTCCAAATTAGC |
