# Supplementary material for: Flax rust infection transcriptomics reveals a transcriptional profile that may be indicative for rust Avr genes
Source: PLoS One. 2019 Dec 12;14(12):e0226106. doi: 10.1371/journal.pone.0226106 (PMC6907798; doi:10.1371/journal.pone.0226106)
Supplement: S2 Table — (DOCX) [file pone.0226106.s005.docx]

**S2 Table.** **Amplicon information for candidate reference genes.**

| Gene | *M. lini* ortholog locus | Protein | Amplicon size (bp) | Amplification efficiency* | *R*^2^ * |
| --- | --- | --- | --- | --- | --- |
| *TUB1* | MELLI_sc591.2 | β-tubulin | 147 | 1.778 | 0.9965 |
| *GAPDH* | MELLI_sc5387.1 | Glyceraldehyde-3-phosphate dehydrogenase | 273 | 1.971 | 0.9969 |
| *SNOG408* | MELLI_sc421.6 | Hypothetical protein SNOG10408 | 216 | 1.941 | 0.9921 |

* The qPCR amplification efficiency and correlation coefficients (*R*^2^) were calculated for PCR products at 3-8 dpi using the LinRegPCR program [46] and averaged between three biological replicates.
