# Supplementary material for: Flax rust infection transcriptomics reveals a transcriptional profile that may be indicative for rust Avr genes
Source: PLoS One. 2019 Dec 12;14(12):e0226106. doi: 10.1371/journal.pone.0226106 (PMC6907798; doi:10.1371/journal.pone.0226106)
Supplement: S3 Table — (DOCX) [file pone.0226106.s006.docx]

**S3 Table.** **RNA-Seq lane allocations and resulting read data for 24 cDNA libraries (three biological replicates of eight samples) of flax (L. usitatissimum, cultivar Hoshangabad) leaves uninoculated or inoculated with flax rust (*M. lini,* strain CH5).^a^**

| Lane | Replicate  number | Sample description | Number of reads | Data yield (bp) |
| --- | --- | --- | --- | --- |
| 1 | 1 | Uninoculated flax leaves | 29,721,778 | 1.49 Gb |
|  | 2 | Uninoculated flax leaves | 32,172,295 | 1.61 Gb |
|  | 3 | Uninoculated flax leaves | 30,972,360 | 1.55 Gb |
|  | 1 | 6 h *in vitro* germinated spores | 33,232,858 | 1.66 Gb |
|  | 2 | 6 h *in vitro* germinated spores | 34,008,983 | 1.70 Gb |
|  | 3 | 6 h *in vitro* germinated spores | 32,931,034 | 1.65 Gb |
| 2 | 1 | 2 dpi flax leaves | 91,661,628 | 4.58 Gb |
|  | 2 | 2 dpi flax leaves | 95,963,861 | 4.80 Gb |
| 3 | 3 | 2 dpi flax leaves | 93,765,076 | 4.69 Gb |
|  | 1 | 3 dpi flax leaves | 99,383,523 | 4.97 Gb |
| 4 | 2 | 3 dpi flax leaves | 96,951,941 | 4.85 Gb |
|  | 3 | 3 dpi flax leaves | 95,134,782 | 4.76 Gb |
| 5 | 1 | 4 dpi flax leaves | 60,951,303 | 3.05 Gb |
|  | 2 | 4 dpi flax leaves | 67,890,733 | 3.39 Gb |
|  | 3 | 4 dpi flax leaves | 62,035,735 | 3.10 Gb |
| 6 | 1 | 5 dpi flax leaves | 62,747,007 | 3.14 Gb |
|  | 2 | 5 dpi flax leaves | 61,601,760 | 3.08 Gb |
|  | 3 | 5 dpi flax leaves | 65,477,369 | 3.27 Gb |
| 7 | 1 | 6 dpi flax leaves | 31,549,496 | 1.58 Gb |
|  | 2 | 6 dpi flax leaves | 32,917,514 | 1.65 Gb |
|  | 3 | 6 dpi flax leaves | 30,092,557 | 1.50 Gb |
|  | 1 | 8 dpi flax leaves | 29,814,379 | 1.49 Gb |
|  | 2 | 8 dpi flax leaves | 31,451,896 | 1.57 Gb |
|  | 3 | 8 dpi flax leaves | 33,161,578 | 1.66 Gb |

^a^Sequence data (50 bp single-end reads) were generated using the Illumina CASAVA pipeline, version 1.8.2, at the AGRF (Melbourne, VIC, Australia).
