# Supplementary material for: Flax rust infection transcriptomics reveals a transcriptional profile that may be indicative for rust Avr genes
Source: PLoS One. 2019 Dec 12;14(12):e0226106. doi: 10.1371/journal.pone.0226106 (PMC6907798; doi:10.1371/journal.pone.0226106)
Supplement: S1 File — (DOCX) [file pone.0226106.s010.docx]

**S1 File. References.**

88. Cañero DC, Roncero MIG. Functional analyses of laccase genes from *Fusarium oxysporum*. Phytopathology. 2008;98:509-518.

89. Fernández IS, Ruíz-Dueñas FJ, Santillana E, Ferreira P, Martínez MJ, Martínez ÁT, et al. Novel structural features in the GMC family of oxidoreductases revealed by the crystal structure of fungal aryl-alcohol oxidase. Acta Crystallographica. Section D, Biological Crystallography. 2009;65:1196-1205.

90. Zámocký M, Hallberg M, Ludwig R, Divne C, Haltrich D. Ancestral gene fusion in cellobiose dehydrogenases reflects a specific evolution of GMC oxidoreductases in fungi. Gene. 2004;338:1-14.

91. Singh D, Chen S. The white-rot fungus *Phanerochaete chrysosporium*: conditions for the production of lignin-degrading enzymes. Appl Microbiol Biotechnol. 2008;81:399-417.

92. Lee M-H, Lai W-L, Lin S-F, Hsu C-S, Liaw S-H, Tsai Y-C. Structural characterization of glucooligosaccharide oxidase from *Acremonium strictum*. Appl Environ Microbiol. 2005;71:8881-8887.

93. Caufrier F, Martinou A, Dupont C, Bouriotis V. Carbohydrate esterase family 4 enzymes: substrate specificity. Carbohydr Res. 2003;338:687-692.

94. Kanneganti V, Gupta AK. Isolation and expression analysis of *Os*PME1, encoding for a putative pectin methyl esterase from *Oryza sativa* (subsp. *indica*). Physiol Mol Biol Plants. 2009;15:123-131.

95. Itoh T, Ochiai A, Mikami B, Hashimoto W, Murata K. A novel glycoside hydrolase family 105: the structure of family 105 unsaturated rhamnogalacturonyl hydrolase complexed with a disaccharide in comparison with family 88 enzyme complexed with the disaccharide. J Mol Biol. 2006;360:573-585.

96. Stam MR, Danchin EGJ, Rancurel C, Coutinho PM, Henrissat B. Dividing the large glycoside hydrolase family 13 into subfamilies: towards improved functional annotations of α-amylase-related proteins. Protein Eng Des Sel. 2006;19:555-562.

97. Sauer J, Sigurskjold BW, Christensen U, Frandsen TP, Mirgorodskaya E, Harrison M, et al. Glucoamylase: structure/function relationships, and protein engineering. Biochim Biophys Acta. 2000;1543:275-293.

98. Kotake T, Hirata N, Degi Y, Ishiguro M, Kitazawa K, Takata R, et al. Endo-β-1,3-galactanase from winter mushroom *Flammulina velutipes*. J Biol Chem. 2011;286:27848-27854.

99. Ferrer P, Halkier T, Hedegaard L, Savva D, Diers I, Asenjo JA. Nucleotide sequence of a β-1, 3-glucanase isoenzyme IIA gene of *Oerskovia xanthineolytica* LL G109 (*Cellulomonas cellulans*) and initial characterization of the recombinant enzyme expressed in *Bacillus subtilis*. J Bacteriol. 1996;178:4751-4757.

100. Ashida H, Maskos K, Li S-C, Li Y-T. Characterization of a novel endo-β-galactosidase specific for releasing the disaccharide GlcNAcα1->4Gal from glycoconjugates. Biochemistry. 2002;41:2388-2395.

101. Gaudioso-Pedraza R, Benitez-Alfonso Y. A phylogenetic approach to study the origin and evolution of plasmodesmata-localized glycosyl hydrolases family 17. Frontiers in Plant Science. 2014;5:212.

102. Lopes MA, Gomes DS, Koblitz MGB, Pirovani CP, Cascardo JCdM, Góes-Neto A, et al. Use of response surface methodology to examine chitinase regulation in the basidiomycete *Moniliophthora perniciosa*. Mycol Res. 2008;112:399-406.

103. Seidl V, Huemer B, Seiboth B, Kubicek CP. A complete survey of *Trichoderma* chitinases reveals three distinct subgroups of family 18 chitinases. The FEBS Journal. 2005;272:5923-5939.

104. Nascimento AS, Muniz JRC, Aparício R, Golubev AM, Polikarpov I. Insights into the structure and function of fungal β-mannosidases from glycoside hydrolase family 2 based on multiple crystal structures of the *Trichoderma harzianum* enzyme. The FEBS Journal. 2014;281:4165-4178.

105. Intra J, Pavesi G, Horner DS. Phylogenetic analyses suggest multiple changes of substrate specificity within the Glycosyl hydrolase 20 family. BMC Evol Biol. 2008;8:214.

106. Taylor EJ, Goyal A, Guerreiro CIPD, Prates JAM, Money VA, Ferry N, et al. How family 26 glycoside hydrolases orchestrate catalysis on different polysaccharides: structure and activity of a *Clostridium thermocellum* lichenase, *Ct*Lic26A. The Journal of Biological Chemistry. 2005;280:32761-32767.

107. Hart DO, He S, Chany CJⅡ, Withers SG, Sims PFG, Sinnott ML, et al. Identification of Asp-130 as the catalytic nucleophile in the main α-galactosidase from *Phanerochaete chrysosporium*, a family 27 glycosyl hydrolase. Biochemistry. 2000;39:9826-9836.

108. Li B, Renganathan V. Gene cloning and characterization of a novel cellulose-binding β-glucosidase from *Phanerochaete chrysosporium*. Appl Environ Microbiol. 1998;64:2748-2754.

109. Ernst HA, Lo Leggio L, Willemoës M, Leonard G, Blum P, Larsen S. Structure of the *Sulfolobus solfataricus* α-glucosidase: implications for domain conservation and substrate recognition in GH31. J Mol Biol. 2006;358:1106-1124.

110. Tanthanuch W, Chantarangsee M, Maneesan J, Ketudat-Cairns J. Genomic and expression analysis of glycosyl hydrolase family 35 genes from rice (*Oryza sativa* L.). BMC Plant Biol. 2008;8:84.

111. Morrison JM, Elshahed MS, Youssef N. A multifunctional GH39 glycoside hydrolase from the anaerobic gut fungus *Orpinomyces* sp. strain C1A. PeerJ. 2016;4:e2289.

112. Mewis K, Lenfant N, Lombard V, Henrissat B. Dividing the large glycoside hydrolase family 43 into subfamilies: a motivation for detailed enzyme characterization. Appl Environ Microbiol. 2016;82:1686-1692.

113. Tremblay LO, Herscovics A. Cloning and expression of a specific human α 1,2-mannosidase that trims Man_9_GlcNAc_2_ to Man_8_GlcNAc_2_ isomer B during *N*-glycan biosynthesis. Glycobiology. 1999;9:1073-1078.

114. Aspeborg H, Coutinho PM, Wang Y, Brumer HⅢ, Henrissat B. Evolution, substrate specificity and subfamily classification of glycoside hydrolase family 5 (GH5). BMC Evol Biol. 2012;12:186.

115. Jackson CL. Arf proteins and their regulators: at the interface between membrane lipids and the protein trafficking machinery. In: Wittinghofer A, editor. Ras Superfamily Small G Proteins: Biology and Mechanisms 2: Springer, Cham; 2014. pp. 151-180.

116. Tefsen B, Lagendijk E, Park J, Akeroyd M, Schachtschabel D, Winkler R, et al. Fungal α-arabinofuranosidases of glycosyl hydrolase families 51 and 54 show a dual arabinofuranosyl- and galactofuranosyl-hydrolyzing activity. Biol Chem. 2012;393:767-775.

117. Miyazaki T, Matsumoto Y, Matsuda K, Kurakata Y, Matsuo I, Ito Y, et al. Heterologous expression and characterization of processing β-glucosidase I from *Aspergillus brasiliensis* ATCC 9642. Glycoconj J. 2011;28:563-571.

118. Voutilainen SP, Puranen T, Siika‐Aho M, Lappalainen A, Alapuranen M, Kallio J, et al. Cloning, expression, and characterization of novel thermostable family 7 cellobiohydrolases. Biotechnol Bioeng. 2008;101:515-528.

119. Yoshimi A, Miyazawa K, Abe K. Function and biosynthesis of cell wall α-1, 3-glucan in fungi. Journal of Fungi. 2017;3:63.

120. Mouyna I, Fontaine T, Vai M, Monod M, Fonzi WA, Diaquin M, et al. Glycosylphosphatidylinositol-anchored glucanosyltransferases play an active role in the biosynthesis of the fungal cell wall. The Journal of Biological Chemistry. 2000;275:14882-14889.

121. Maddi A, Fu C, Free SJ. The *Neurospora crassa* *dfg5* and *dcw1* genes encode α-1,6-mannanases that function in the incorporation of glycoproteins into the cell wall. PLoS One. 2012;7:e38872.

122. Kutty G, Davis AS, Ma L, Taubenberger JK, Kovacs JA. *Pneumocystis* encodes a functional endo-β-1,3-glucanase that is expressed exclusively in cysts. The Journal of Infectious Diseases. 2015;211:719-728.

123. Lastovetsky OA, Gaspar ML, Mondo SJ, LaButti KM, Sandor L, Grigoriev Ⅳ, et al. Lipid metabolic changes in an early divergent fungus govern the establishment of a mutualistic symbiosis with endobacteria. Proceedings of the National Academy of Sciences. 2016;113:15102-15107.

124. Mahajan S, Master ER. Proteomic characterization of lignocellulose-degrading enzymes secreted by *Phanerochaete carnosa* grown on spruce and microcrystalline cellulose. Appl Microbiol Biotechnol. 2010;86:1903-1914.

125. Lombard V, Bernard T, Rancurel C, Brumer H, Coutinho PM, Henrissat B. A hierarchical classification of polysaccharide lyases for glycogenomics. The Biochemical Journal. 2010;432:437-444.

126. Michaud P, Pheulpin P, Petit E, Séguin JP, Barbotin JN, Heyraud A, et al. Identification of glucuronan lyase from a mutant strain of *Rhizobium meliloti*. Int J Biol Macromol. 1997;21:3-9.

127. Downie B, Gurusinghe S, Dahal P, Thacker RR, Snyder JC, Nonogaki H, et al. Expression of a galactinol synthase gene in tomato seeds is up-regulated before maturation desiccation and again after imbibition whenever radicle protrusion is prevented. Plant Physiol. 2003;131:1347-1359.
